# 

**Published:** 1913-01

**Authors:** 


### CONTENTS.

ORIGINAL COMMUNICATIONS:—

The Relation of the Eye to General Disease. By Cecil Stockard, M. D., Atlanta, Ga. ........ 503

Diphtheria; Some Observations After Inspection of 22,000 School Children For Two Successive Years. By Claude A. Smith, M. D., Atlanta, Ga. ........ 506

The Control of Typhoid Infection. By Charles T. Nesbit, M. D., Superintendent of Health, Wilmington, N. C. ........ 509

Hernias of the Uterine Appendages. By Aime Paul Heineck, M. D., Surgeon to the Cook County Hospital, Chicago. ........ 512

A New Theory as to the Etiology of Pellagra. By Roy Blosser, M. D., Atlanta, Ga. ........ 527

Pituitary Extract as an Ecbolic. By F. F. Dollert, Associate Professor of Obstetrics in the Medical Department of Marquette University, Etc., Milwaukee, Wis. ........ 531

EDITORIAL ........ 539

SELECTIONS AND ABSTRACTS ........ 543

BOOK REVIEWS ........ 549

